# Backpropagation With Sparsity Regularization for Spiking Neural Network Learning

**DOI:** 10.3389/fnins.2022.760298

**Published:** 2022-04-14

**Authors:** Yulong Yan, Haoming Chu, Yi Jin, Yuxiang Huan, Zhuo Zou, Lirong Zheng

**Affiliations:** School of Information Science and Technology, Fudan University, Shanghai, China

**Keywords:** spiking neural network, backpropagation, sparsity regularization, spiking sparsity, synaptic sparsity

## Abstract

The spiking neural network (SNN) is a possible pathway for low-power and energy-efficient processing and computing exploiting spiking-driven and sparsity features of biological systems. This article proposes a sparsity-driven SNN learning algorithm, namely backpropagation with sparsity regularization (BPSR), aiming to achieve improved spiking and synaptic sparsity. Backpropagation incorporating spiking regularization is utilized to minimize the spiking firing rate with guaranteed accuracy. Backpropagation realizes the temporal information capture and extends to the spiking recurrent layer to support brain-like structure learning. The rewiring mechanism with synaptic regularization is suggested to further mitigate the redundancy of the network structure. Rewiring based on weight and gradient regulates the pruning and growth of synapses. Experimental results demonstrate that the network learned by BPSR has synaptic sparsity and is highly similar to the biological system. It not only balances the accuracy and firing rate, but also facilitates SNN learning by suppressing the information redundancy. We evaluate the proposed BPSR on the visual dataset MNIST, N-MNIST, and CIFAR10, and further test it on the sensor dataset MIT-BIH and gas sensor. Results bespeak that our algorithm achieves comparable or superior accuracy compared to related works, with sparse spikes and synapses.

## 1. Introduction

Artificial intelligence (AI) has shown impressive abilities in various tasks such as computer vision, natural language processing, and decision making. For example, AlphaGo Zero defeated the world champion of the game of Go (Silver et al., 2017). However, the power consumption of AlphaGo Zero is about 1kW (Frenkel et al., 2021), which is 50× higher than the 20W power budget of the human brain (Roy et al., 2019). The brain-inspired spiking neural network (SNN) plays an important role in addressing the issue of AI energy efficiency. SNN exchanges information through binary spikes between synapses and performs intensive calculation only when spikes are received. Dedicated SNN hardware such as TrueNorth (Akopyan et al., 2015), Loihi (Davies et al., 2018), Tianjic (Pei et al., 2019), and MindWare (Ding et al., 2021) can reduce energy consumption from sparse spikes and synapses through spike-driven computing architecture. Despite the merits of improving energy efficiency, there remain a lot of challenges ahead of the SNN in sparsity learning algorithms and efficient network exploration.

The commonly adopted SNN learning algorithms can be summarized into three different types as follows. (1) *Conversion-based learning*. It uses the same SNN structure as an artificial neural network (ANN) and converts the parameters of the learned ANN to SNN. One conversion idea is to use the spiking firing rate (FR) of SNN to quantify the floating value of ANN and establish an approximate mapping between the parameters of two networks (Sengupta et al., 2019; Kim et al., 2020). This kind of conversion uses rate coding, resulting in dense spikes. Another idea is to use spike timing to represent the floating value in ANN. Methods like time-to-first-spike (TTFS) conversion (Rueckauer and Liu, 2018) and few spikes conversion (FS-conversion) (Stöckl and Maass, 2021) use temporal coding to protect spiking sparsity. However, the time domain is used for coding so that temporal processing structure such as recurrent neural network (RNN) cannot be converted. (2) *Plasticity-based learning*. It is a kind of biologically inspired algorithm. The most famous spike-timing-dependent plasticity (STDP) adjusts synaptic weight according to the spike order between the pre- and post-synaptic neurons. The role of STDP is feature clustering. Combined with lateral inhibition structure, STDP can realize unsupervised classification (Diehl and Cook, 2015; Białas and Mańdziuk, 2021). Reward-modulated STDP draws on the eligibility trace of reinforcement learning to realize supervised learning to further improve performance (Mozafari et al., 2018). The plasticity-based learning algorithm is skilled in computation overhead and weak in network accuracy. (3) *Gradient-based learning*. Like the learning of ANN, it updates the parameters of SNN according to the gradient information from backpropagation. A recent study by Lillicrap et al. (2020) suggests that a similar propagation mechanism may exist in the brain. Spatio-temporal backpropagation (STBP) (Wu et al., 2018, 2019) provides advanced accuracy by calculating gradient in the spatio-temporal domain. Deep continuous local learning (DECOLLE) (Kaiser et al., 2020) reduces the memory overhead through the local error function. Spike-train level recurrent SNN backpropagation (ST-RSBP) (Zhang and Li, 2019) further supports the recurrent layer, to deal with temporal information by mimicking import feedback structure in the brain (Luo, 2021). The above algorithms focus on the accuracy improvement and lack consideration in the sparsity issue. Compared with local learning based on plasticity, gradient-based learning requires global information. It improves accuracy and brings additional calculation burdens. However, in the offline learning scenario, the computational overhead of SNN is mainly contributed by inference rather than learning. Therefore, reducing the computational overhead in inference through sparsity optimization and ensuring accuracy by gradient-based learning, become the major motivation of this work.

Another kind of SNN algorithm aims to improve synaptic sparsity by pruning. Existing studies explore different pruning standards. Liang et al. (2021) prune synapses through random patterns and quantify synaptic weight to reduce storage overhead. Rathi et al. (2018) utilize the synaptic weight threshold to prune and optimize storage through weight quantization and sharing. Cho et al. (2019) prune long-range synaptic connections based on the small world theory of the nervous system. Nguyen et al. (2021) combine pruning with STDP and use the weight adjustment record as the pruning standard. Shi et al. (2019) use spiking count as the pruning threshold and propose a soft pruning method to reduce the computation overhead in learning. Moreover, Guo et al. (2020) prune the neurons rather than synapses according to spiking count, providing a new perspective of sparsity exploration.

SNN can perform sparse computing due to the event-driven feature. At the same time, the synaptic operation uses membrane potential accumulation instead of matrix multiplication and addition in traditional ANN, which reduces the amount of calculation. In recent years, similar methods have been proposed in the field of ANN to reduce the number of operations. Binarized neural network (BNN) (Hubara et al., 2016) and XNOR-Net (Rastegari et al., 2016) introduce binarized weights and activations and replace most arithmetic operations on synapses with bit-wise operations. AdderNet (Chen et al., 2020) builds ANN only through addition to avoid the expensive multiplication operation and achieves acceleration with low energy consumption. Beyond that, Bartol et al. (2015) believe each synapse stores about 4.7 bits of information. Quantization of synaptic weights can also be an idea to further optimize computational speed and compress storage overhead.

**Figure 1:**
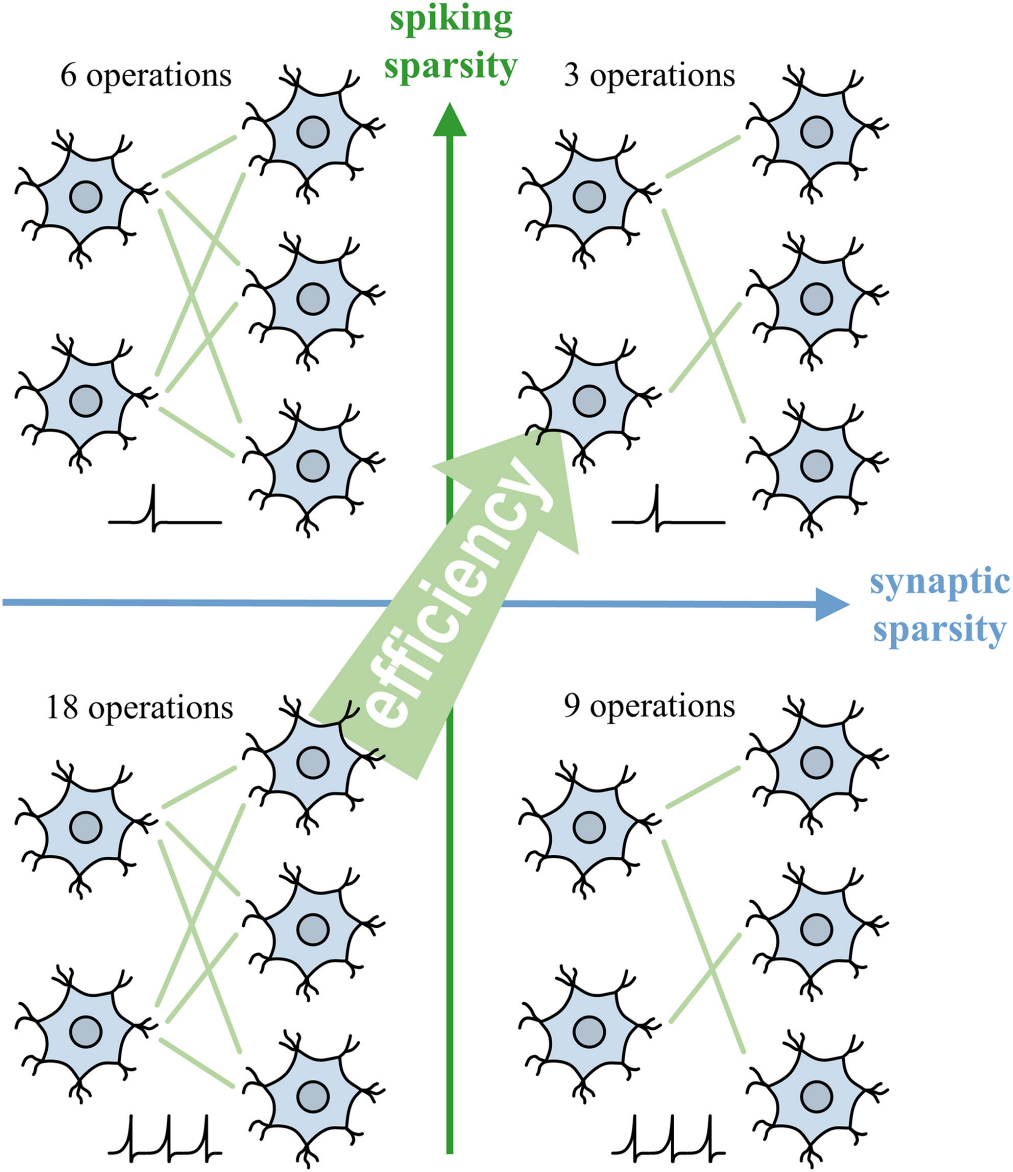
Spiking sparsity and synaptic sparsity facilitate the efficiency of SNN by reducing the number of synaptic operations.

This work proposes an SNN learning algorithm, namely backpropagation with sparsity regularization (BPSR) to facilitate sparsity. As shown in Figure 1, the sparse spikes reduce the amount of information that subsequent neurons need to process, meanwhile the sparse synapses prevent each spike from causing intensive calculations. The proposed BPSR enables SNN to improve sparsity during learning and achieve satisfactory energy efficiency in inference. The backpropagation takes advantage of temporal information and adapts the brain-like recurrent structure. BPSR balances the accuracy and FR by combining backpropagation with spiking regularization. Inspired by the fact that the brain learns through synaptic rearrangement (Dempsey et al., 2022), rewiring mechanism is proposed to explore efficient SNN structures, which uses the weight and gradient to regulate synaptic pruning and growth. The experimental result is consistent with the concept that the proposed BPSR can achieve low FR with high accuracy. Spiking sparsity is proved to be beneficial to SNN learning (Tang et al., 2017), because of the suppression of information redundancy. BPSR not only improves the synaptic sparsity but also generates a bionic structure similar to the nervous system of *Caenorhabditis elegans* (*C. elegans*). The result on the visual MNIST dataset (LeCun et al., 1998) with rank order coding (Thorpe and Gautrais, 1998), neuromorphic-MNIST (N-MNIST) (Orchard et al., 2015), and CIFAR10 (Krizhevsky et al., 2009) reach the accuracy of 98.33, 99.21, and 90.74%, respectively. The evaluation on MNIST also shows 30× the inference overhead advantage compared to other SNN works. With post-training quantization (PTQ), SNN can achieves 15× efficiency compared to BNN with 0.22% accuracy drop. BPSR is further tested on sensor datasets like MIT-BIH arrhythmia (Moody and Mark, 2001) and gas senor (Vergara et al., 2013), which achieves 98.41 and 98.30% accuracy.

The remainder of this article is organized as follows. In Section 2, the backpropagation with sparsity regularization is introduced. The suggested heterogeneous neuron dynamic model, the loss function with regularization, and the backpropagation algorithm on the flat and recurrent SNN layers are detailed. In Section 3, the rewiring based on weight and gradient and the corresponding implementation process is introduced. In Section 4, the effect of the proposed BPSR algorithm is tested by experiments, and comparisons with related works on various datasets are reported. In Section 5, we summarize this work and make a discussion.

## 2. Backpropagation With Sparsity Regularization

The backpropagation algorithm with regularization updates SNN parameters while improving sparsity. The spiking sparsity is implemented through backpropagation and spiking regularization. Synaptic sparsity requires the cooperation of regularization and the rewiring mechanism in Section 3. Firstly, a heterogeneous leaky integrate-and-fire (LIF) neuron dynamic model and its differential approximation are suggested. Secondly, a classification loss function with spiking regularization and synaptic regularization is introduced. Finally, the backpropagation algorithm for the flat SNN layer and the brain-like recurrent SNN layer is detailed, respectively.

### 2.1. Heterogeneous Leaky Integrate-and-Fire Model

**Figure 2:**
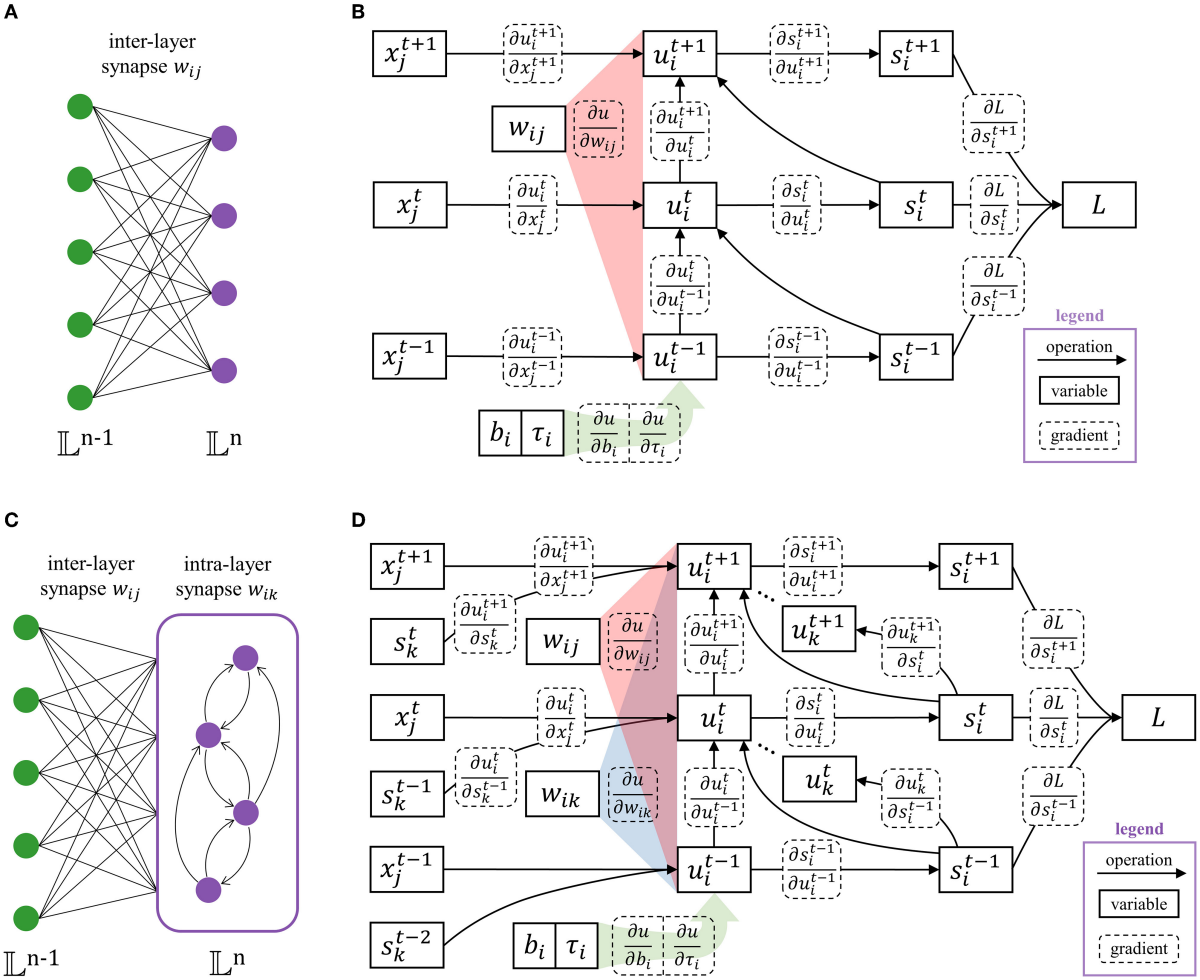
The structure of the SNN layer contains **(A)** the flat layer with only inter-layer synapses (*w*_*ik*_ = 0), and **(C)** the recurrent layer with intra-layer synapses (*w*_*ij*_ ≠ 0). The corresponding computational graphs are **(B,D)**, respectively. The legends of arithmetic operations, neuron state variables, and gradients are marked in the lower right corner.

As one of the most commonly used neuron models, LIF describes the dynamic process of neurons in SNN. The membrane potential of neurons increases under the stimulation of spikes and leaks spontaneously with time. When the potential reaches the spiking threshold, the neuron generates a spike and resets the membrane potential. In addition, we extend the LIF description to the spiking recurrent layer and support neurons with different time coefficients (heterogeneous), to utilize the brain-like structure and temporal features. We hierarchically describe the SNN. For the *n*-th layer, the LIF process can be described by equations in the discrete-time domain:


(1) $$\begin{array}{l} u_{i}^{t}=u_{i}^{t-1}\cdot \tau_{i}\cdot \bar{s_{i}^{t-1}}+\underset{j\in {𝕃}^{\text{n}-1}}{\sum }w_{ij}\cdot x_{j}^{t}+\underset{k\in {𝕃}^{\text{n}}}{\sum }w_{ik}\cdot s_{k}^{t-1}+b_{i},i\in {𝕃}^{\text{n}} \end{array}$$

(2) $$\begin{array}{l} s_{i}^{t}=g\left(u_{i}^{t}-U_{th}\right) \end{array}$$

where $u_{i}^{t}$ is the membrane potential of *i*-th neuron in layer 𝕃^n^ at time *t* (𝕃^n^ represents the set of neurons in the *n*-th layer). $s_{i}^{t}\in \left\{0,1\right\}$ is a boolean value where $s_{i}^{t}=1$ denotes a spike activity. $\bar{s_{i}^{t-1}}$ means to take a logical ‘not' operation on $s_{i}^{t-1}$. τ_*i*_ ∈ [0, 1] is the leakage time coefficient, which achieves neuronal heterogeneity. This allows the neuron model to be heterogeneous and facilitates temporal feature extracting. Multiply $u_{i}^{t-1}$ by $\tau_{i}\cdot \bar{s_{i}^{t-1}}$ controls whether the membrane potential leaks by τ_*i*_ or drops to the resting potential 0. The neuron bias is denoted by *b*_*i*_, leading to self-excitation or self-suppression. $x_{j}^{t}$ is the input spike from the *j*-th neuron in layer 𝕃^n−1^. It should be noted that, for the calculation of layer 𝕃^n+1^, $x:=s_{i}^{t},i\in {𝕃}^{\text{n}}$. In this way, spikes are transmitted layer by layer. As shown in Figure 2, the SNN layer can be classified as Figure 2A the flat layer and Figure 2C the recurrent layer. For the flat layer, *w*_*ij*_ represents inter-layer synapse from the *j*-th neuron in layer 𝕃^n−1^ to *i*-th neuron in layer 𝕃^n^. For the recurrent, *w*_*ik*_ is appended to indicate intra-layer synapse inside layer 𝕃^n^, which has the ability to extract temporal features due to the brain-like structure. The Heaviside function *g*(·) generates a spike when $u_{i}^{t}$ is greater than or equal to the spiking threshold *U*_*th*_. Heaviside function and the adopted differential approximation are expressed as:


(3) $$\begin{array}{l} g\left(x\right)=\{\begin{array}{cc} 1, & \;x\geq 0\\ 0, & \;x<0 \end{array}\;,\;g^{\prime }\left(x\right)=\frac{\alpha }{\sqrt{\pi }}e^{-\alpha^{2}x^{2}} \end{array}$$

Backpropagation requires a differentiable path. The derivative of the Heaviside function *g*(·) is the Dirac function δ(·), whose value is +∞ at 0 and impossible to perform the calculation. Thus, the Gaussian function is introduced as the differential approximation of the Heaviside function, where α controls the shape of the function.

### 2.2. Loss Function With Sparsity Regularization

The loss function measures the error for a classification task and the sparsity of SNN, which is defined as follows. The first term measures the classification error through softmax and cross-entropy functions. The second and third terms achieve spiking sparsity and synaptic sparsity, respectively.


(4) $$\begin{array}{l} L=\underset{\text{classification error}}{\underset{︸}{-\underset{c}{\sum }y_{c}\log\left(p_{c}\right)}}+\underset{\text{spiking sparsity}}{\underset{︸}{\frac{\lambda_{s}}{2}\underset{i\notin {𝕃}^{\text{N}}}{\sum }\underset{t}{\sum }{\left\|s_{i}^{t}\right\|}_{2}^{2}}}+\underset{\text{synaptic sparsity}}{\underset{︸}{\lambda_{w}\underset{w\in {𝕃}^{\text{n}}}{\sum }{\left\|w\right\|}_{1}}} \end{array}$$

(5) $$\begin{array}{l} p_{c}=\text{softmax}\left(k\cdot \underset{t}{\sum }s_{c}^{t}\right)=\frac{\exp\left(\sum_{t}k\cdot s_{c}^{t}\right)}{\sum_{i\in {𝕃}^{\text{N}}}\exp\left(\sum_{t}k\cdot s_{i}^{t}\right)} \end{array}$$

where *y*_*c*_ is the ground-truth label of one-hot coding for the *c*-th class. *p*_*c*_ is the predicted probability given by the output layer 𝕃^N^. *p*_*c*_ is calculated by summing of the output spikes, multiplied by factor *k*, and then processing by softmax function. The factor $k=\frac{10}{T}$ corrects the softmax error by scaling the sum of spikes in the time window *T*. λ_*s*_ is the coefficient of *l*_2_ regularization for spiking sparsity. It takes effect on the spikes of the SNN layer, except for the output layer to ensure classification accuracy. λ_*w*_ is the coefficient of *l*_1_ regularization for the sparsity of synaptic weight, which is effective for all layers of the SNN.

The regularizations of spiking sparsity and synaptic sparsity have similar forms and can promote each other. But in essence, their mechanism is different (as shown in Table 1). The goal of spiking regularization is to reduce FR while ensuring guaranteed accuracy. Therefore, the regular term adjusts the parameters *w*_*ij*_, *w*_*ik*_, *b*_*i*_, and τ_*i*_ to punish dense spikes. Synaptic regularization works together with the rewiring mechanism in Section 3 to realize pruning of the weight *w*_*ij*_ and *w*_*ik*_. The gradient of spiking regularization is calculated by the chain rule. When either input spike $x_{j}^{t}$ or output spike $s_{i}^{t}$ is 0, the spiking regularization will not be affected. The gradient of synaptic regularization is calculated directly, and it works continuously until the weight is set to 0. As regularization, both of them improve network performance by preventing overfitting. The difference is that the principle of spiking regularization is simplifying feature expression to improve generalization ability. While synaptic regularization and rewiring work together to take effect by reducing the dimensionality of the parameter space.

**Table 1:** Comparison between spiking and synaptic regularization.

**Regularization**	**Purpose**	**Scope**	**Gradient of synaptic weight**
Spiking	Reduce FR while ensuring accuracy	*w*_*ij*_, *w*_*ik*_, *b*_*i*_, τ_*i*_	$\nabla_{w}\propto \lambda_{s}\cdot s_{i}^{t}g^{\prime }\left(u_{i}^{t}\right)x_{j}^{t}$
Synaptic	Combine with rewiring for pruning	*w*_*ij*_, *w*_*ik*_	∇_*w*_ = λ_*w*_ · sign(*w*)

### 2.3. Backpropagation in Flat Layer

The error information is propagated through the gradient and the parameters of SNN are updated accordingly. Therefore, it is necessary to derive the gradient of the loss function to each parameter. There are only inter-layer synaptic connections in the flat layer structure and *w*_*ik*_ = 0. The computational graph of the flat layer and the corresponding gradient path are shown in Figure 2B. For the output layer 𝕃^N^, the partial derivative ∂*L*/∂*s* can be directly calculated. For the non-output layer (𝕃^n^, *n* < *N*), the partial derivative ∂*L*/∂*s* is the ∂*L*/∂*x* of the following layer, plus the spiking regularization term.


(6) $$\begin{array}{l} \frac{\partial L}{\partial s_{i}^{t}}=\{\begin{array}{l} \;p_{i}-y_{i},\;i\in {𝕃}^{\text{N}}\\ \frac{\partial L}{\partial x_{j}^{t}}+\lambda_{s}\cdot x_{j}^{t},\;i\in {𝕃}^{\text{n}},\;j\in {𝕃}^{\text{n}+1},\;n<N \end{array} \end{array}$$

The spike $s_{i}^{t}$ is a function of the membrane potential $u_{i}^{t}$, and the membrane potential changes over time. Although $u_{i}^{t}$ is a function of $s_{i}^{t-1}$ in Equation (1), $s_{i}^{t-1}$ only gates the information flow in potential along time. Unlike $u_{i}^{t}$ accumulating information to $s_{i}^{t}$, or $x_{j}^{t-1}$ passing *w*_*ij*_ of information to $u_{i}^{t}$, $s_{i}^{t-1}$ has no information contribution to $u_{i}^{t}$. Thus, $\partial u_{i}^{t}/\partial s_{i}^{t-1}$ is ignored in backpropagation. $\partial L/\partial u_{i}^{t}$ is expressed as:


(7) $$\begin{array}{l} \frac{\partial L}{\partial u_{i}^{t}}=\{\begin{array}{l} \frac{\partial L}{\partial s_{i}^{t}}\cdot \frac{\partial s_{i}^{t}}{\partial u_{i}^{t}}=\frac{\partial L}{\partial s_{i}^{t}}\cdot g^{\prime }\left(u_{i}^{t}-U_{th}\right),\;t=T\\ \frac{\partial L}{\partial s_{i}^{t}}\cdot \frac{\partial s_{i}^{t}}{\partial u_{i}^{t}}+\frac{\partial L}{\partial u_{i}^{t+1}}\cdot \frac{\partial u_{i}^{t+1}}{\partial u_{i}^{t}}\\ =\frac{\partial L}{\partial s_{i}^{t}}\cdot g^{\prime }\left(u_{i}^{t}-U_{th}\right)+\frac{\partial L}{\partial u_{i}^{t+1}}\cdot \tau_{i}\cdot \bar{s_{i}^{t}},\;t<T \end{array} \end{array}$$

**Figure 3:**
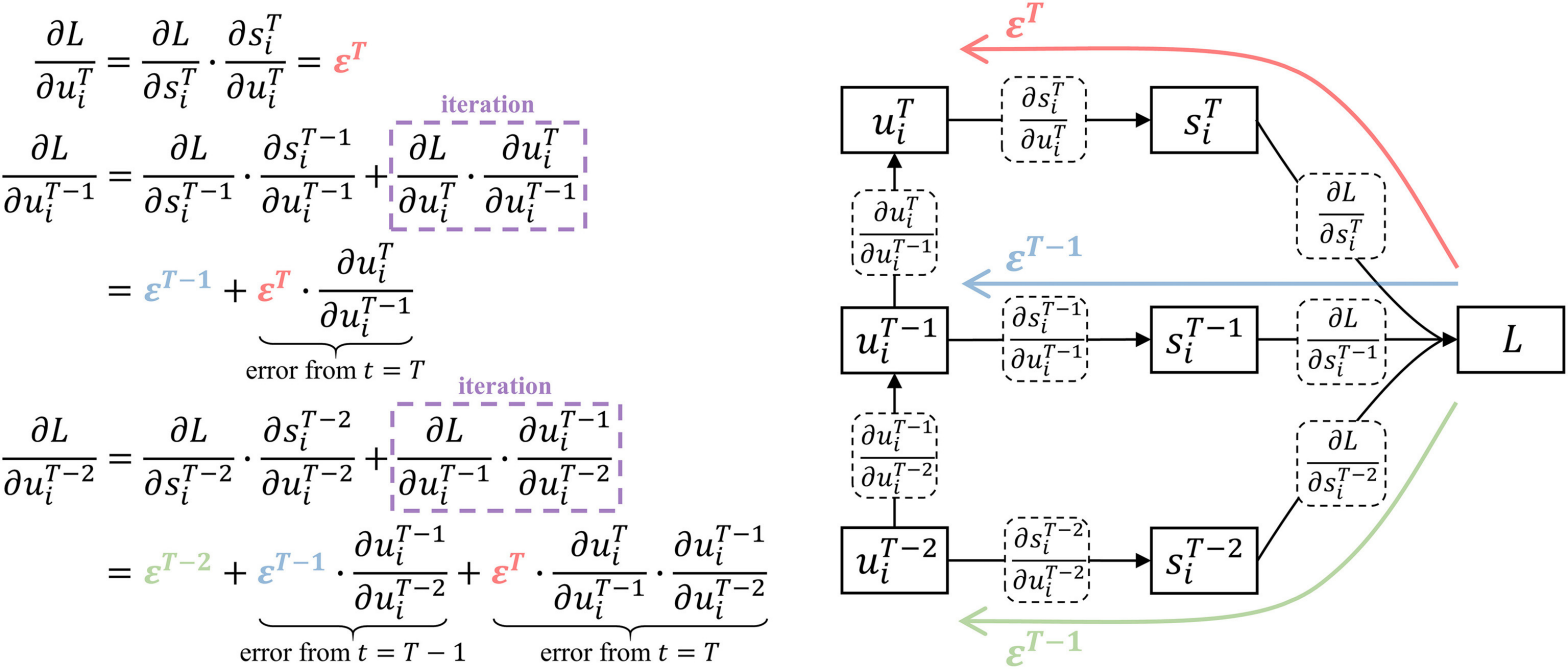
Iterative calculation with linear algorithm complexity. At time *T*, the potential error comes from the direct error ε^*T*^. At time *T* − 1, the potential error includes the direct error ε^*T*−1^ and the backpropagation of ε^*T*^. At time *T* − 2, the influence of ε^*T*^, ε^*T*−1^, ε^*T*−2^ are taken into account through iterative calculation, which only requires one addition and multiplication.

The part of *t* < *T* in Equation (7) takes into account all the errors after time *t* through iterative calculation and reduces the algorithm complexity to *O*(*t*). Assuming the direct error from the loss function at time *t* is $\varepsilon^{t}=\partial L/\partial s_{i}^{t}\cdot \partial s_{i}^{t}/\partial u_{i}^{t}$. Figure 3 shows how the influence from the subsequent time is calculated by one addition and multiplication when *t* = *T, T*−1, *T*−2.

Once the gradient to $u_{i}^{t}$ is obtained, the gradients to each parameter and input spike are easy to calculate by the following equations, where *i* belongs to layer 𝕃^n^ and *T* is the time window. The initial value $u_{i}^{0}=s_{i}^{0}=0$. Learning shared parameters such as convolution weights or homogeneous leakage coefficients can be realized by summing the gradient of shared weight. Potential changes well beyond the threshold have no effect, so excessively large *w*_*ij*_ and *b*_*i*_ are meaningless and clamped to [−*U*_*th*_, +*U*_*th*_] accordingly. τ is also limited to its range of values [0, 1].


(8) $$\begin{array}{l} \frac{\partial L}{\partial x_{j}^{t}}=\underset{i\in {𝕃}^{\text{n}}}{\sum }\frac{\partial L}{\partial u_{i}^{t}}\cdot \frac{\partial u_{i}^{t}}{\partial x_{j}^{t}}=\underset{i\in {𝕃}^{\text{n}}}{\sum }\frac{\partial L}{\partial u_{i}^{t}}\cdot w_{ij} \end{array}$$

(9) $$\begin{array}{l} \frac{\partial L}{\partial w_{ij}}=\overset{T}{\underset{t=1}{\sum }}\frac{\partial L}{\partial u_{i}^{t}}\cdot \frac{\partial u_{i}^{t}}{\partial w_{ij}}+\lambda_{w}\cdot \text{sign}\left(w_{ij}\right)\\ \;=\overset{T}{\underset{t=1}{\sum }}\frac{\partial L}{\partial u_{i}^{t}}\cdot x_{j}^{t}+\lambda_{w}\cdot \text{sign}\left(w_{ij}\right) \end{array}$$

(10) $$\begin{array}{l} \frac{\partial L}{\partial b_{i}}=\overset{T}{\underset{t=1}{\sum }}\frac{\partial L}{\partial u_{i}^{t}}\cdot \frac{\partial u_{i}^{t}}{\partial b_{i}}=\overset{T}{\underset{t=1}{\sum }}\frac{\partial L}{\partial u_{i}^{t}} \end{array}$$

(11) $$\begin{array}{l} \frac{\partial L}{\partial \tau_{i}}=\overset{T}{\underset{t=1}{\sum }}\frac{\partial L}{\partial u_{i}^{t}}\cdot \frac{\partial u_{i}^{t}}{\partial \tau_{i}}=\overset{T}{\underset{t=1}{\sum }}\frac{\partial L}{\partial u_{i}^{t}}\cdot u_{i}^{t-1}\cdot \bar{s_{i}^{t-1}} \end{array}$$

### 2.4. Backpropagation in Recurrent Layer

The intra-layer synaptic connections exist in the recurrent layer, i.e., *w*_*ik*_ ≠ 0. This makes the computational graph of the recurrent layer and the gradient path are different from the flat layer, which are shown in Figure 2D. The calculation method of the partial derivative $\partial L/\partial s_{i}^{t}$ still follows Equation (6). Considering the intra-layer connection within the recurrent, $\partial L/\partial u_{i}^{t}$ is modified to:


(12) $$\begin{array}{l} \frac{\partial L}{\partial u_{i}^{t}}=\{\begin{array}{l} \frac{\partial L}{\partial s_{i}^{t}}\cdot \frac{\partial s_{i}^{t}}{\partial u_{i}^{t}}=\frac{\partial L}{\partial s_{i}^{t}}\cdot g^{\prime }\left(u_{i}^{t}-U_{th}\right),\;t=T\\ \frac{\partial L}{\partial s_{i}^{t}}\cdot \frac{\partial s_{i}^{t}}{\partial u_{i}^{t}}+\frac{\partial L}{\partial u_{i}^{t+1}}\cdot \frac{\partial u_{i}^{t+1}}{\partial u_{i}^{t}}+\underset{k\in {𝕃}^{\text{n}}}{\sum }\frac{\partial L}{\partial u_{k}^{t+1}}\cdot \frac{\partial u_{k}^{t+1}}{\partial s_{i}^{t}}\cdot \frac{\partial s_{i}^{t}}{\partial u_{i}^{t}}\\ =\left[\frac{\partial L}{\partial s_{i}^{t}}+\underset{k\in {𝕃}^{\text{n}}}{\sum }\frac{\partial L}{\partial u_{k}^{t+1}}\cdot w_{ki}\right]\cdot g^{\prime }\left(u_{i}^{t}-U_{th}\right)\\ +\frac{\partial L}{\partial u_{i}^{t+1}}\cdot \tau_{i}\cdot \bar{s_{i}^{t}},t<T \end{array} \end{array}$$

Note that for the intra-layer synaptic weight, we swap the subscripts of the input and the output neurons (denoted as *w*_*ki*_). The above equation is still an iterative calculation with time complexity of *O*(*t*). The calculation of the gradient of each parameter is still consistent with Equation (8)–(9). As a supplement, ∂*L*/∂*w*_*ik*_ can be calculated by the following equation, where *i* and *k* both belong to layer 𝕃^n^ and the initial value $s_{k}^{0}=0$.


(13) $$\begin{array}{l} \frac{\partial L}{\partial w_{ik}}=\overset{T}{\underset{t=1}{\sum }}\frac{\partial L}{\partial u_{i}^{t}}\cdot \frac{\partial u_{i}^{t}}{\partial w_{ik}}+\lambda_{w}\cdot \text{sign}\left(w_{ik}\right)\\ \;=\overset{T}{\underset{t=1}{\sum }}\frac{\partial L}{\partial u_{i}^{t}}\cdot s_{k}^{t-1}+\lambda_{w}\cdot \text{sign}\left(w_{ik}\right) \end{array}$$

In this way, the required gradients are obtained. Errors can be passed down layer by layer. Each network parameter can be updated by various general ANN parameter optimization algorithms, such as stochastic gradient descent (SGD), adaptive momentum estimation (Adam) (Kingma and Ba, 2014) or Adam with decoupled weight decay (AdamW) (Loshchilov and Hutter, 2017).

### 2.5. Post-training Quantization

Fixed-point quantification can compress the storage overhead of SNN, and achieve higher computational efficiency by replacing floating-point arithmetic with fixed-point arithmetic. We use PTQ to quantify parameters, avoiding the overhead of re-learning. After learning, PTQ quantizes *w* and *b* into *n*-bit fixed-point numbers, where the fraction length is *n* - 1 and the signedness is 1-bit. This allows synaptic operations to be performed through fixed-point addition instead of floating-point addition. τ is rounded to 2^−*m*^, so that the multiplication on the potential is replaced by *m*-bit right shift operation. PTQ brings optimization of storage overhead and energy consumption under the condition of limited accuracy loss.

## 3. Rewiring Based on Weight and Gradient

**Figure 4:**
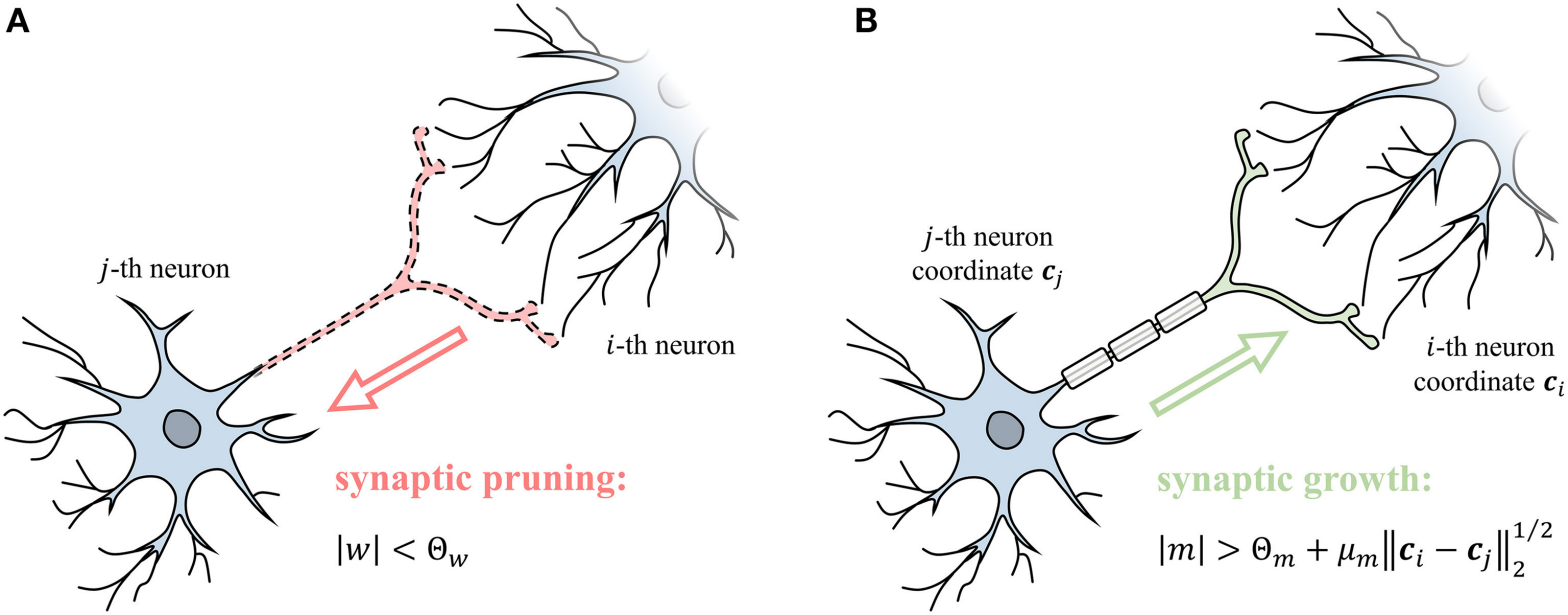
**(A)** The weight of synapse *w* controls the synaptic pruning. **(B)** The momentum of the synapse gradient *m* controls the synaptic growth.

Rewiring mechanism prunes and grows synapses based on synaptic weights and gradients to improve synaptic sparsity. Synaptic weights are constantly decreasing in learning through synaptic regularization. When the |*w*| is less than the pruning threshold Θ_*w*_ (Equation 14), it means that the influence on the post-synaptic neuron is negligible and synapse can be pruned (Figure 4A). Moreover, the pruned synapses have a chance to reconnect through growth. The gradient of the synaptic weight represents a trend of growth. The momentum *m* is the exponential moving averaging of the synaptic gradient ∇_*w*_, where β_*m*_ is the coefficient of moving average. The *m* measures the strength of the growth trend after smoothing fluctuations. When the *m* is large enough to satisfy Equation (15), the synapse grows as shown in Figure 4B. The growth conditions include a constant threshold Θ_*m*_ and a distance term scaled by the ratio μ_*m*_, where ***c***_*i*_ and ***c***_*j*_ represent the spatial coordinates of two neurons. The above condition means that establishing a longer-range synaptic connection requires a stronger growth trend. Dynamic rewiring is coupled with the learning process, using pruning and growth to improve sparsity and ensure performance. SNN is finally stable between rewiring and parameter optimization and acquires a sparse and efficient network structure.


(14) $$\begin{array}{l} \text{pruning }:\;|w|<\Theta_{w} \end{array}$$

(15) $$\begin{array}{l} \text{growth }:\;|m|>\Theta_{m}\cdot \left(1+\mu_{m}{\left\|c_{i}-c_{j}\right\|}_{2}^{1/2}\right),\;\\ \;m\;:=m+\left(1-\beta_{m}\right)\nabla_{w} \end{array}$$

The rewiring mechanism works together with backpropagation and parameter optimization. The pseudo-code (Algorithm 1) takes layer 𝕃^n^ as an example to illustrate how to implement the proposed BPSR algorithm with matrix operation. The input spike matrix **X** and the gradient matrix of output spike **Δ**_S_ are required. $N^{n}$ represents the number of neurons in layer 𝕃^n^ and *T* is the time window. The shape of **X** is $N^{n-1}\times T$. The gradient **Δ**_S_ with shape of $N^{n}\times T$ can be backpropagated by the following layer through Equation (6). The notation [*t*] is used to represent the matrix slice in the time dimension. The algorithm generates the output spike **S** and the gradient of the input spike **Δ**_X_, and ensures to update the synaptic weight matrix **W**, bias matrix **B** and, leakage coefficient matrix T. For the flat layer, we mark the synaptic weight as **W** = **W**_*ij*_. For the recurrent layer, the synaptic weight matrix is the concatenation **W** = [**W**_*ij*_|**W**_*ik*_]. In the initial stage, the weight matrix **W** is set to obey Gaussian distribution N(0,1). The bias matrix **B** is initialized to uniform distribution U(0,1). The leakage coefficient matrix T is set to an empirical value of 0.5. The coordinates of neurons **C** are set to the random distribution in the unit cube. Especially, Kaiming initialization (He et al., 2015) is applied to the convolutional layer. The coordinates of neurons **C** are set to the random distribution in the unit cube. The forward and backpropagation processes are described in the previous sections. In the rewiring, **Prun** and **Grow** are two boolean matrices, denoting the synapses that meet the conditions 14 and 15. The boolean matrix **Mask** indicates the existing synapses after rewiring. Logical operations “and” and “or” achieve prune and grow, respectively. The **W** and **Δ**_W_ is superimposed by **Mask**. Finally, all parameters are updated through the ANN optimization algorithm and clamped.

**Algorithm 1:** The BPSR implementation of layer 𝕃^n^.

**Require:** Input spike **X**. The gradient of output spike **Δ**_S_ obtained by backpropagation.
**Ensure:** Output spike **S**. The gradient of input spike **Δ**_X_. Update parameters **W**, **B** and T.
*Initialization*:
1: W←N(0,1), B←U(0,1), T←0.5, *U*_*th*_ ← 1, C←U(0,1) // Initialize if applicable. *Forward*:
2: **for** *t* = 1 to *T* **do**
3: U[t]←CalU(U[t-1],S[t-1],X[t],W,B,T) // Calculate potential by Equation (1). Specially **S**[0] = 0.
4: **S**[*t*] ← CalS(**U**[*t*]) // Calculate spike by Equation (2).
5: **end for**
*Backpropagation*:
6: // Calculate gradient of potential by Equation (7) and (12).
7: **Δ**_U_[*T*] ← CalG_U_(**Δ**_S_[*t*], **U**[*t*])
8: **for** *t* = *T* − 1 to 1 **do**
9: $\Delta_{\text{U}}\left[t\right]\leftarrow \text{Cal}{\text{G}}_{\text{U}}\left(\Delta_{\text{S}}\left[t\right],\Delta_{\text{U}}\left[t+1\right],\text{U}\left[t\right],\text{S}\left[t\right],T\right)$
10: **end for**
11: **Δ**_X_ ← CalG_X_(**Δ**_U_, **W**) // Calculate gradient of input spike by Equation (8).
12: **Δ**_W_ ← CalG_W_(**Δ**_U_, **S**, **X**, **W**), **Δ**_B_ ← CalG_B_(**Δ**_U_), $\Delta_{T}\leftarrow \text{Cal}{\text{G}}_{T}\left(\Delta_{\text{U}},\text{U},\text{S}\right)$ // Calculate gradient of parameters by Equations (10)–(9) and (13).
*Rewiring*:
13: M ← CalM(**Δ**_W_, M) // Calculate gradient momentum by Equation (15).
14: Prun ← CalPrun(W), Grow ← CalGrow(M, **Coor**) // Pruning and growth by Equations (14)–(15).
15: $\text{Mask}=\left(\text{W}!=0\right)\;\text{and}\;\bar{\text{Prun}}\;\text{or}\;\text{Grow}$ // Calculate mask of synapse by logical operation.
16: W : = Mask · W, **Δ**_W_: = Mask · **Δ**_W_ // Mask the weight and gradient.
*Updating*:
17: Update **W**, **B** and T with optimization algorithm such SGD, Adam or AdamW.
18: **W** ∈ [−*U*_*th*_, +*U*_*th*_], **B** ∈ [−*U*_*th*_, +*U*_*th*_], T∈[0,1] // Clamp parameters.

## 4. Experimental Results

**Figure 5:**
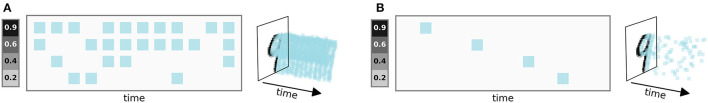
The principle of **(A)** rate coding and **(B)** rank order coding, and the spike sequence of an MNIST image after coding.

The proposed BPSR is implemented by PyTorch (Paszke et al., 2019) and runs on a CPU of AMD Ryzen-3970X and a GPU of NVIDIA RTX-3080. Various visual datasets and sensor datasets are used in the experiments. MNIST is a static digital dataset and can be transformed into a spiking dataset by rate coding and rank order coding. Rate coding (Figure 5A) takes pixel intensity as the probability and performs Bernoulli sampling in the time domain to produce spikes. Rank order coding (Figure 5B) convert higher values to earlier spikes, which is a kind of temporal sparse coding. Unlike rate coding, the spiking timing in rank order is meaningful. This requires the SNN to have the capacity for temporal processing. N-MNIST is a spiking version of MNIST and is acquired by the dynamic vision sensor (DVS). It is widely used in SNN research due to event-driven and neuromorphic. CIFAR10 is another static visual dataset for object classification of color images. We employ the encoding layer proposed by Wu et al. (2019) to convert floating values to spikes. MIT-BIH is an arrhythmia dataset that includes 48 sets of electrocardiographs (ECG). The level-crossing (LC) sampling (Marisa et al., 2017) converts signal into spike. 2-channel ECG generates 4-channel spiking input suitable for SNN, as shown in Section 4.1. The gas sensor dataset is the record from a chemical detection platform in a wind tunnel facility in response to ten high-priority chemical gaseous substances. The 72-channel sensing signal is encoded by rank order to obtain the spiking input.

### 4.1. Coding Method and Feature Visualization

**Figure 6:**
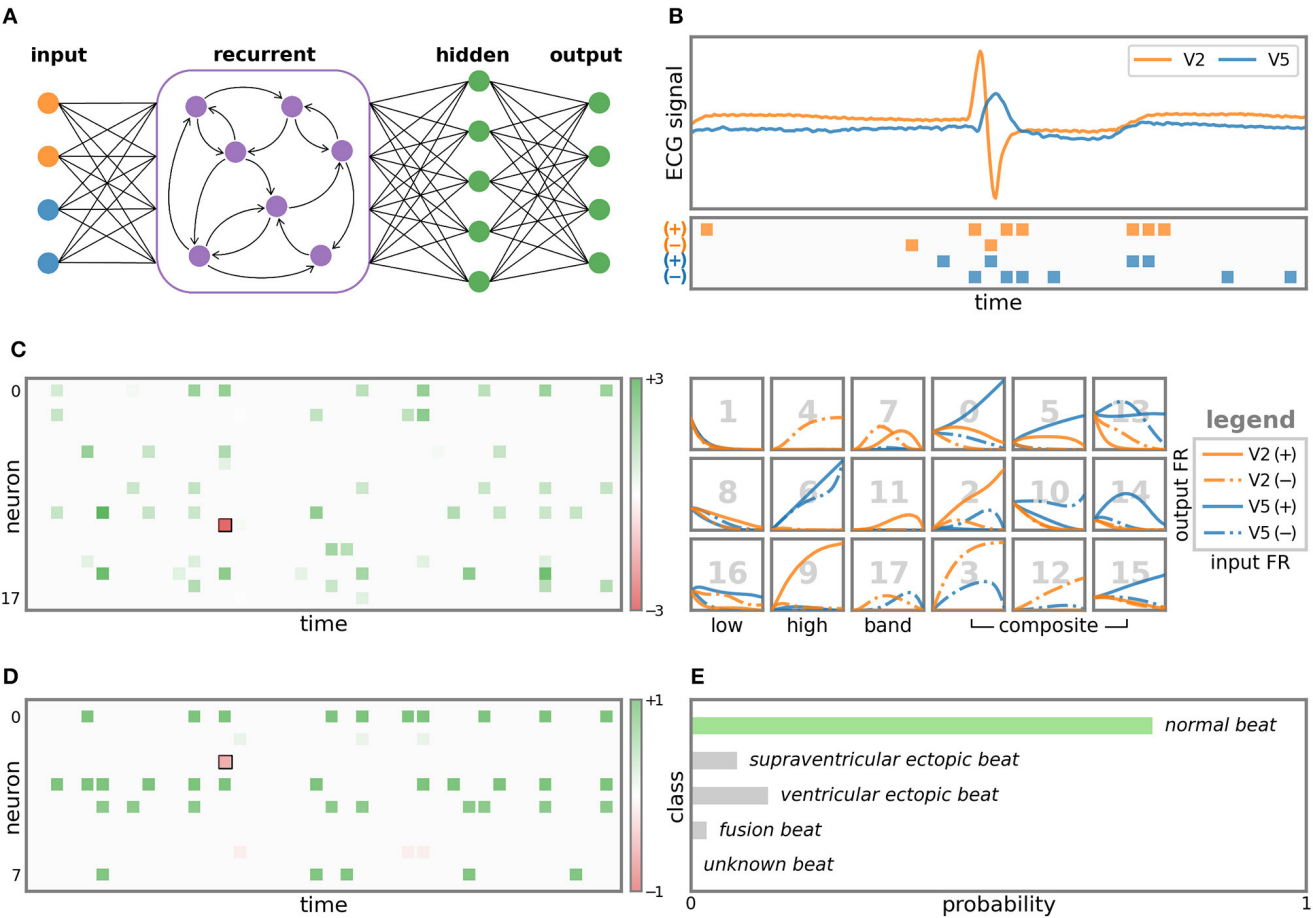
Visualization of LC sampling and each layer of SNN. **(A)** The structure of the SNN model. **(B)** The ECG signal and the input spikes after LC sampling. **(C)** Output spikes and corresponding FR response curves of 18 neurons in the recurrent layer. The coordinates of the spikes represent the occurrence time and the neuron index. The color indicates the impact on the prediction result, where green is positive and red is negative. Response curves are plotted channel-by-channel. The x-axis and y-axis are the input and output FR, respectively. The 18 neurons are classified according to the filter effect, and the corresponding neuron index is marked in gray number. The output spikes of the hidden layer are drawn on **(D)**, and the predicted probability for the 5 ECG classes is shown on **(E)**.

A 5-class ECG task is used to show how SNN processes temporal information. The SNN model resented in Figure 6A is the recurrent MLP (rMLP) of “*r*18 - *fc*8 - *fc*5”, where “*r*” denotes the recurrent layer and ‘*fc*' denotes the fully connected layer. Figure 6B demonstrates the original ECG signal and the spiking sequence after LC sampling. The 2 channels of the displayed record 102 are modified lead V2 and V5, and other records may contain modified limb lead II (MLII). Bipolar spikes are generated on the edge of signal changes in each channel. In this way, the spike reflects the changing trend of the signal. 4-channel spikes input to the recurrent layer for temporal feature processing. In the right of Figure 6C, the output FR curve of the recurrent layer under different input FR is plotted channel by channel. Neurons can be classified into low-pass, high-pass, band-pass and composite characteristics according to different filter effects. The left of Figure 6C shows the spike output of the recurrent layer and its influence on the prediction result. All neurons have a positive effect (green) on the prediction results, except for neuron 11 marked by the black box. In addition, neurons 0, 10, and 15 make more contributions, revealing that the corresponding frequency features are more important for predicting this class. Figure 6D is the spike output of the hidden layer. The role of this layer is the feature mapping before prediction. All neurons also make a positive effect except for one neuron. The final prediction result (Figure 6E) is the spike sum of 5 output neurons and is normalized to probability. It can be seen that the SNN makes the correct prediction for a normal heartbeat.

### 4.2. Algorithm Efficiency

**Figure 7:**
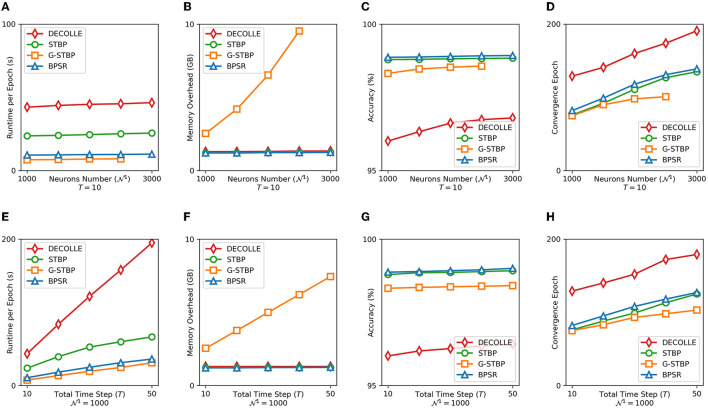
**(A)** Runtime, **(B)** graphic memory overhead, **(C)** accuracy, and **(D)** convergence epoch of four learning algorithms are counted under the different number of hidden layer neurons $N^{1}$. Panels **(E–H)** are the corresponding indicator under the different length of time window *T*.

The runtime and memory overhead reflect the efficiency of the algorithm, the accuracy and convergence epoch number prove its effectiveness. The proposed BPSR is compared with the other three SNN gradient descent algorithms, namely DECOLLE, STBP, and graph-based STBP (G-STBP) (Yan et al., 2021a). The four algorithms are all implemented based on PyTorch and accelerated by the GPU to get a fair comparison. The MNIST is encoded by rate coding as the time window *T* and the learning batch size is set to 32. The SNN model is a three-layer multilayer perceptron (MLP), where the size of the input layer is 784 and the output layer is 10. The number of neurons in the hidden layer ($N^{1}$) is a variable in the experiment. Figure 7 shows the algorithm runtime of a single epoch, the graphic memory overhead on the GPU, the accuracy with rate coding in different situations, and the number of epochs required for SNN learning to reach convergence. It can be seen that G-STBP has the smallest runtime in any case, also accompanied by the highest memory overhead. G-STBP describes the network as a whole adjacency graph. This allows backpropagation to be carried out on the entire network together instead of layer by layer, but the inter-layer connection is expressed as zero resulting in memory overhead. BPSR simplifies the storage and calculation burden of intermediate quantities through iterative calculations, bringing faster runtime (2.1× than STBP) and smaller memory overhead. BPSR also achieves the highest accuracy in all cases, with the second most convergence epoch, verifying its effectiveness.

### 4.3. Spiking Sparsity and Synaptic Sparsity

**Figure 8:**
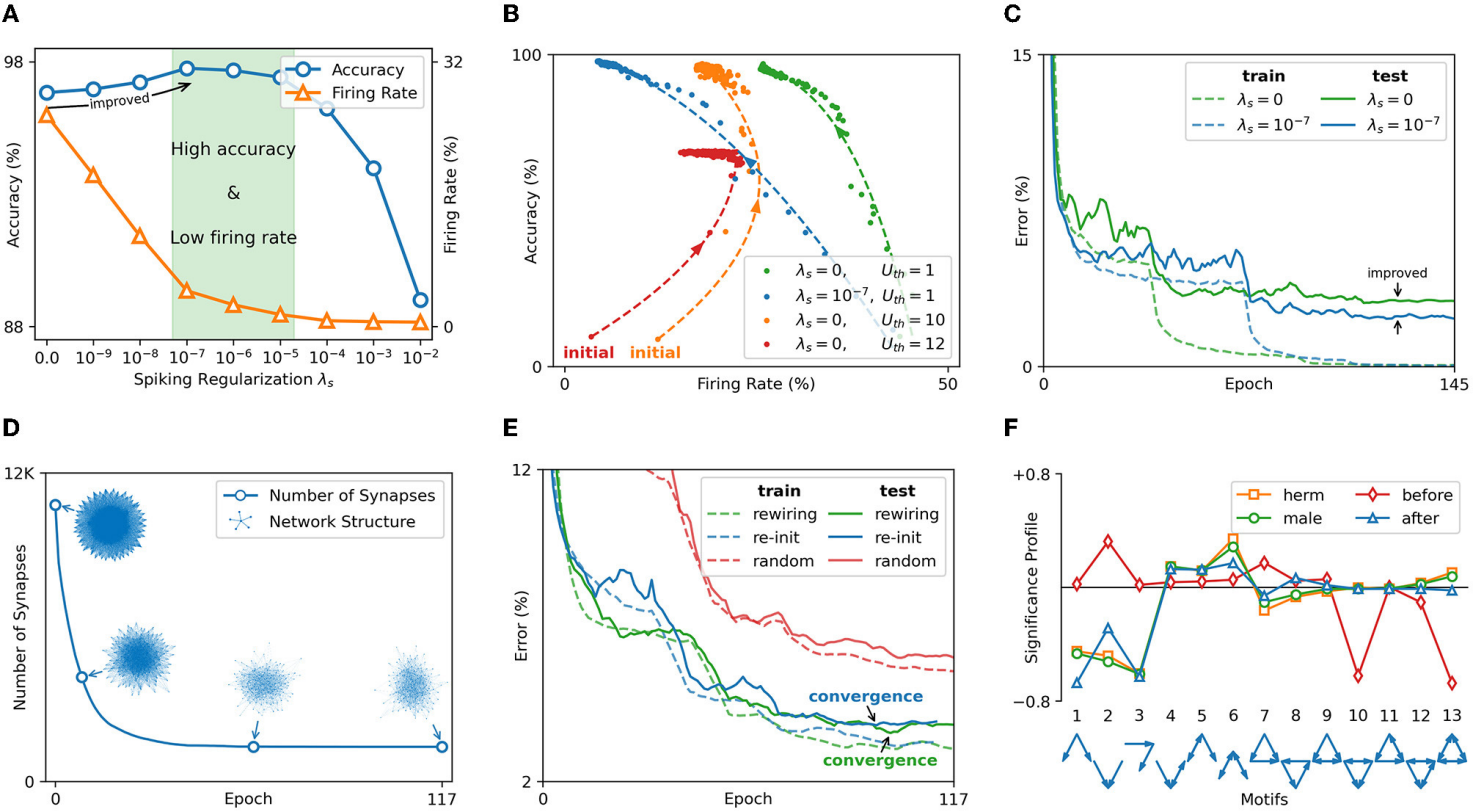
**(A)** The test accuracy and FR under different spiking regularization coefficients λ_*s*_. **(B)** Accuracy and FR change law in the learning process. **(C)** Learning curves under different λ_*s*_ (after smoothing filtering). **(D)** The number of synapses and network structure changes in the recurrent layer. **(E)** The learning curve with and without rewiring mechanism (after smoothing filtering). **(F)** Significance profile of *C. elegans* nervous system and the gas sensor network.

The effect of spiking sparsity regularization is tested on the MNIST dataset encoded by rank order. The used SNN model is rMLP of “*r*1000 - *fc*100 - *fc*10.” The accuracy and average FR of the test set are counted under different spiking regularization coefficients λ_*s*_. The count of FR excludes the input spike because it is controlled by the encoding method rather than the regularization. It can be seen from Figure 8A that FR decreases as the spiking regularization coefficient λ_*s*_ increases. Spiking regularization forces SNN to express information with fewer spikes. Through appropriate λ_*s*_, the SNN can achieve high accuracy with low computation overhead in the inference. Moreover, the accuracy is improved with the decrease of FR when $\lambda_{s}\in \left[0,10^{-7}\right]$. One reason is that SNN learning is a process of FR reduction. As shown in Figure 8B, the accuracy and FR are approximately inversely related during the learning process. SNN learns important features by suppressing redundant information. Setting a high initial threshold (*U*_*th*_ = 10) causes the FR to increase first and then become an inversely proportional learning process. Inappropriately high threshold (*U*_*th*_ = 12) can even lead to network divergence. The learning curve in Figure 8C verifies that spiking regularization can prevent overfitting. Under the same training error, the SNN with spike regularization achieves improved test accuracy and shows better generalization.

The effect of synaptic sparsity regularization is tested on the gas sensor dataset and the learned SNN structure is compared with the nervous system of *Caenorhabditis elegans* (*C. elegans*). The neuron connection graph of *C. elegans* has been fully studied (Cook et al., 2019). The hermaphrodite and the male have 302 neurons and 385 neurons, respectively. 83 sensory neurons and 81 interneurons are the same for all genders. The tested SNN model is “*r*81 - *fc*36 - *fc*10.” The input layer and the first hidden layer have a similar number of neurons as the *C. elegans*, which is convenient for structural comparison. SNN learns under synaptic regularization coefficient λ_*w*_ = 0.01. The line in Figure 8D shows the number of synapses in the input layer and the first hidden layer. The point cloud plots the network structure (topological connection) during the rewiring process. After 117 epochs, the network can be 8× in the recurrent layer. In Figure 8E, the above network obtained by rewiring is re-initialized to evaluate the convergence speed. SNN with the same number of synapses but a random structure is also tested. Experiment shows that the SNN without rewiring will reach the lowest error 4 epochs earlier than the SNN with rewiring. SNN with random structures has higher errors, demonstrating the effect of rewiring.

The efficacy of rewiring is further verified by significance profile (SP) (Milo et al., 2004), a method of analyzing the similarity of network structure. It measures the structural characteristics of the network by comparing the number of occurrences of different induced subgraphs (i.e., motifs) in the network. The possible connection modes between the three nodes are used as 13 motifs. A set of random networks is generated as the reference based on the degree sequence of the network to be tested. The numbers of occurrences of 13 motifs in the network to be tested and the random network set are recorded as the 13-dimensional vector **N**_*test*_ and vector set **N**_*rand*_, respectively. The SP is the vector normalization of $\left({\text{N}}_{test}-{\bar{\text{N}}}_{rand}\right)/\text{std}\left({\text{N}}_{rand}\right)$. The SP of hermaphrodite (herm) and male *C. elegans*, and the SP of SNN before and after learning are plotted in Figure 8F. It can be seen that the hermaphrodite and the male *C. elegans* have the same structural characteristics. After BPSR learning, the structure of SNN is more similar to the nervous system of *C. elegans*, which means that the rewiring mechanism can generate an effective and bionic network structure.

### 4.4. Evaluation of Performance

Table 2 provides the network structure and hyper-parameters used in the various experiments below. Convolutional indicator “8*c*5/2” means kernel size 5, output channel 8 and stride 2. “*r*” and “*fc*” denote the recurrent layer and the fully connected layer, respectively. [·] means a residual block (He et al., 2016). For convolutional neurons, τ is homogeneous and shared while learning. For neurons in other layers, τ is heterogeneous.

**Table 2:** SNN structures and hyper-parameters setup.

**Structure**	
MNIST	8*c*5/2-16*c*3/2 - *r*100 - *fc*10
N-MNIST	4*c*5/2-16*c*3/2-32*c*3 - *r*100 - *fc*10
CIFAR10	$64c7/2\text{-}\left[\begin{array}{c} 128\text{c}3\\ 128\text{c}3 \end{array}\right]\text{-}\left[\begin{array}{c} 256\text{c}3/2\\ 256\text{c}3 \end{array}\right]\text{-}\left[\begin{array}{c} 512\text{c}3/2\\ 512\text{c}3 \end{array}\right]\text{-}\left[\begin{array}{c} 1024\text{c}3/2\\ 1024\text{c}3 \end{array}\right]\text{-}fc1024\text{-}fc10$
MIT-BIH	*r*256 - *fc*96 - *fc*18 (18 classes)
	*r*192 - *fc*64 - *fc*5 (5 classes)
Gas sensor	*r*128 - *fc*64 - *fc*10
**Hyper-parameter**	
Potential threshold	*U*_*th*_ = 1
Leakage coefficient	τ = 0.5 (initial). *Homogeneous* for conv, otherwise *heterogeneous*.
Coefficient of *g*(·)	α = 0.7
Learning rate	CIFAR10: *lr* = 10^−3^, otherwise: 10^−2^
Sparsity coefficient	CIFAR10: $\lambda_{s}=10^{-9}/10^{-8}$, otherwise: 10^−7^; $\lambda_{w}=10^{-2}$
Rewiring parameter	$\Theta_{w}=10^{-2}$; $\Theta_{m}=10^{-4}$; μ_*m*_ = 5; β_*m*_ = 0.99

#### 4.4.1. MNIST Dataset

Table 3 shows the comparison results of the proposed BPSR and related SNN works on the MNIST dataset. The pooling is taken into account of the number of synapses, and shared weight in the convolution is repeatedly added. The introduction of recurrent layers enhances accuracy but brings additional overhead, which is further improved by sparsity regularization. Compared to other sparse networks using pruning, the proposed BPSR acquired the least number of synapses, with the best spiking sparsity except for G-STBP. Floating-point operations (FLOPs) show the computational overhead of SNN in the learning and inference process. Conversion-based algorithm (Diehl et al., 2015) learns parameters through ANN, avoiding the backpropagation in the time window. It has the lowest learning FLOPs and high accuracy (the conversion cost is underlined and only occurs once after learning). Plasticity-based algorithm is generally considered to be efficient due to local learning rules. However, Diehl and Cook (2015) used a large network to improve the accuracy, resulting in the learning burden. Gradient-based algorithms have high backpropagation overhead but also bring performance optimization. Wu et al. (2018) and Yan et al. (2021a) have improved the SNN with the goal of better accuracy and sparser spikes, respectively. The proposed BPSR achieves a low learning overhead due to its extremely sparse network. Moreover, rank order coded data has a higher learning difficulty due to sparse temporal representation. The accuracy of BPSR is only 0.8–1.1% lower than rate coding, with a 30× inference overhead advantage.

**Table 3:** Comparison of different spiking models on MNIST dataset.

	**Coding**	**Pruning**	**Model**	**Synapses**	**Spikes**	**FLOPs** / **sample**		**Accuracy (%)**
						**learning**	**inference**	
Diehl et al. (2015)	Rate	×	MLP	2.4M	10.0K^*^	24.0+7.2M^*^	6.3M^*^	98.6
			CNN	1.4M	14.7K^*^	7.5+2.9M^*^	2.0M^*^	99.1
Diehl and Cook (2015)	Rate	×	rMLP	46.0M	2.3K	74.7M	15.0M	95.0
Wu et al. (2018)	Rate	×	MLP	0.6M	6.7K^*^	78.9M^*^	2.6M^*^	98.89
			CNN	1.4M	41.4K^*^	162.3M^*^	5.1M^*^	99.42
Yan et al. (2021a)	Rank	×	rMLP	0.3M	392	17.3M	84.5K	97.3
Tang et al. (2020)	Rank	×	CNN	0.6M	——— N/A^**^ ———			90.2
Comşa et al. (2021)	Rank	×	MLP	0.3M	——— N/A^**^ ———			97.96
Shi et al. (2019)	Rate	√	MLP	0.2M	——— N/A^**^ ———			94.05
Guo et al. (2020)	Rate	√	rMLP	0.5M	——— N/A^**^ ———			88.71
Liang et al. (2021)	Rank	√	MLP	0.4M	——— N/A^**^ ———			96
**BPSR (this work)**	Rank	×	CNN	**98K**	**859**	**10.1M**	**86.8K**	**97.56**
		×	rCNN	**0.1M**	**2.6K**		**0.19M**	**98.43**
		√	rCNN	**73K**	**542**		**67.6K**	**98.33**

* *The result is estimated based on the open source code*.

** *Data is not available (N/A) due to the lack of experimental result and source code. The bold values mark our metrics for this work*.

**Figure 9:**
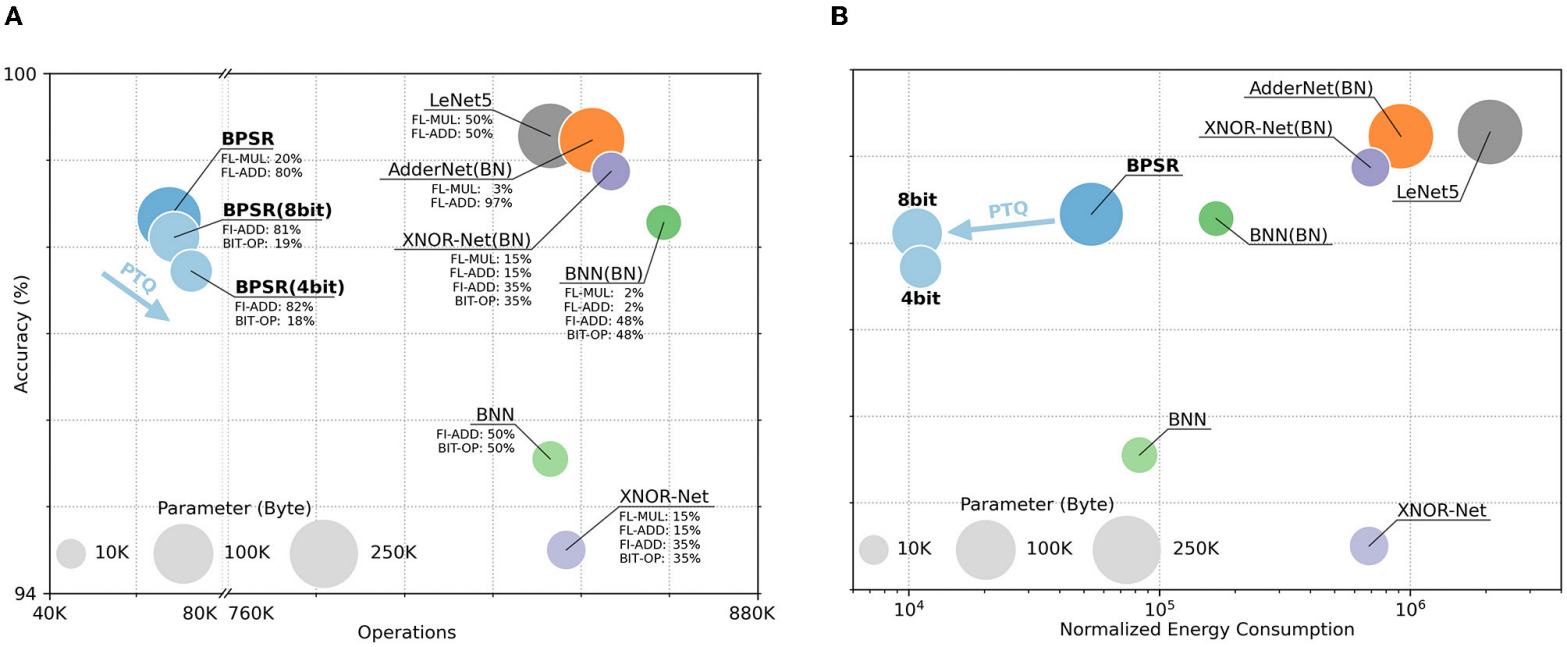
Accuracy, operations, normalized energy consumption, and parameter size of different networks. The area of the circle represents the storage overhead of the parameters. The y-coordinate of the center represents the network accuracy. The x-coordinate represents **(A)** the number of operations and **(B)** the energy consumption of each inference. The proportions of different operations are marked in **(A)**. Additionally, the x-axis of (a) is folded and the x-axis of **(B)** is logarithmic.

Networks such as BNN and AdderNet improve energy efficiency by reducing computational overhead, which is similar to SNN. We also compare the performance of the proposed BPSR and other ANN in Figure 9. The network structure used is LeNet5 and their variant. As mentioned in the original work, batch normalization (BN) (Ioffe and Szegedy, 2015) is introduced to improve accuracy. The involved operations include floating-point multiplication (FL-MUL), floating-point addition (FL-ADD), fixed-point addition (FI-ADD), and bitwise operation (BIT-OP). The network energy consumption in inference is counted by normalization. FL-ADD is considered as unit overhead. FL-MUL is estimated to be 4× of floating-point addition (Cheng et al., 2019), and FI-ADD is estimated to be 20% of FL-ADD (Finnerty and Ratigner, 2017). The overhead of BIT-OP is negligible.

Under the same structure, AdderNet reduces the computational cost by approximating multiplication by addition. BNN and XNOR-Net further reduce storage burden and energy overhead through bitwise operations. The proposed BPSR achieves optimized energy consumption through lightweight structure and sparse spike while ensuring accuracy. PTQ quantizes the parameters of SNN to 8-bit or 4-bit, and further uses fixed-point addition and bitwise right shift instead of floating-point addition and floating-point multiplication to reduce the energy cost. After PTQ, the proposed BPSR reaches 15 ~ 60× energy efficiency than BNN or XNOR-Net, with a 0.22–0.61% accuracy drop of unquantized SNN.

#### 4.4.2. N-MNIST Dataset

Table 4 shows the comparison of N-MNIST. Event-driven N-MNIST is usually converted to frame-based data. Time step *T* matches the time length of the frame sequence. Most networks take few time steps, except Kaiser et al. (2020) which uses 60 steps to warm up the network and 240 steps to learn and infer. Kaiser et al. (2020) uses a shallow network, but the readout layer followed by each regular layer greatly increases the synaptic overhead. The network used by Vaila et al. (2019) is a mixture of ANN and SNN, and the prediction results are given by SVM. Wu et al. (2019) uses the deepest network and most synapses to get the best accuracy. BPSR has minimal synaptic overhead and achieves the second-best accuracy. Introducing a recurrent layer improves the accuracy in the case where the number of tiny synapses grows, proving that the recurrent structure is useful for frame sequence processing.

**Table 4:** Comparison of different spiking models on N-MNIST dataset.

	**Model**	**T**	**Synapses**	**Accuracy (%)**
Wu et al. (2018)	MLP	30	1.9M	98.78
Jin et al. (2018)	MLP	N/A^*^	1.9M	98.93
Wu et al. (2019)	CNN	30	202.4M	99.53
Vaila et al. (2019)	Mixed CNN + SVM	N/A^*^	0.98M	98.32
Kaiser et al. (2020)	CNN	300	315.5M	99.04
**BPSR (this work)**	CNN	20	**0.26M**	**99.15**
	rCNN		**0.26M**	**99.21**

* *Data is not available (N/A) due to the lack of result reports. The bold values mark our metrics for this work*.

#### 4.4.3. CIFAR10 Dataset

We applied the residual SNN on CIFAR10 to verify the performance of the BPSR on the deep model. Table 5 compares BPSR with other SNN works. Sengupta et al. (2019) achieves the best accuracy on VGG16 with a conversion-based learning algorithm. However, the conversion takes 2500 time steps to rate encoding, much higher than other methods. Wu et al. (2019) uses a gradient-based learning algorithm to achieve high accuracy while keeping small time steps. Although in the work of Allred et al. (2020), the accuracy of SNN is limited by the network size, the sparsity resulting from regularization is further explored. We test BPSR on an 11-layer residual network composed of 4 residual blocks. The number of synapses is less than that of other deep networks. The proposed BPSR can reach 90.24% accuracy with the same number of spikes as Allred et al. (2020), or achieve the accuracy of 90.74% with 50% additional spike overhead.

**Table 5:** Comparison of different spiking models on CIFAR10 dataset.

	**Model**	**T**	**Synapses**	**Spikes**	**Accuracy (%)**
Cao et al. (2015)	5-layer CNN	400	5.7M	N/A^*^	77.43
Wu et al. (2018)	4-layer CNN	N/A^*^	2.9M	N/A^*^	50.7
Wu et al. (2019)	8-layer CNN	12	519.8M	N/A^*^	90.53
Sengupta et al. (2019)	VGG16	2500	315.5M	N/A^*^	91.55
Allred et al. (2020)	LeNet5	N/A^*^	0.66M	89.9K	66.45
**BPSR (this work)**	11-layer ResNet	12	**260.7M**	**136.1K** ($\lambda_{s}=10^{-9}$)	**90.74**
		8		**89.6K** ($\lambda_{s}=10^{-8}$)	**90.24**

* *Data is not available (N/A) due to the lack of result reports. The bold values mark our metrics for this work*.

#### 4.4.4. MIT-BIH Dataset

Table 6 are the comparison results between BPSR and related spiking models on the MIT-BIH dataset. Most of the work introduces recurrent structures such as lateral inhibition to process temporal signals. In addition, Kolağasioğlu (2018) use wavelet transform for signal preprocessing, Wu et al. (2020) adopt the gated recurrent unit (GRU), and Corradi et al. (2019) use the support vector machine (SVM) for prediction. These make the implementation no longer pure SNN. MIT-BIH dataset contains various ECG arrhythmia types with a long-tailed distribution. The classification of the fewer sample has a higher learning difficulty. Most works achieve 2-5 classification tasks by selecting subsets and merging certain classes. Kolağasioğlu (2018) and Corradi et al. (2019) take 17 or 18 classes for fine-grained classification. Thus, we used the two models 18 classes and 5 classes. BPSR can make inferences from the compressed time window (*T* = 40), which is more efficient. The proposed BPSR achieves the highest accuracy in fine-grained classification and coarse-grained classification. With the proposed sparsity regularization, the learned models under different classification tasks both achieve optimal synaptic sparsity.

**Table 6:** Comparison of different spiking models on MIT-BIH dataset.

	**Model**	**T**	**Synapses**	**Accuracy (%)**
Kolağasioğlu (2018)	wavelet + rMLP	N/A^*^	N/A^*^	95.5 (17 classes)
Corradi et al. (2019)	rMLP + SVM	250	25.6K	95.6 (18 classes)
Amirshahi and Hashemi (2019)	rMLP	300	968.0K	97.9 (4 classes)
Bauer et al. (2019)	rMLP	N/A^*^	34.8K	97.3 (2 classes)
Wu et al. (2020)	GRU + MLP	N/A^*^	20.8K	97.8 (5 classes)
Yan et al. (2021b)	CNN	180	184.3K	90 (4 classes)
**BPSR (this work)**	rMLP	40	**15.3K**	**97.82 (18 classes)**
			**10.4K**	**98.41 (5 classes)**

* *Data is not available (N/A) due to the lack of result reports*.

#### 4.4.5. Gas Sensor Dataset

Table 7 shows the comparison results of BPSR and related works on the gas sensor dataset. Vergara et al. (2013) use the SVM method to obtain high accuracy. Imam and Cleland (2020) implement the spiking method on Loihi through the external plexiform layer (EPL) structure. Although this method does not perform well in network accuracy, the reported results show high robustness and biological inspiration. BPSR achieves better accuracy and synaptic overhead than related works. At the same time, the proposed SNN with sparsity regularization only needs 762 spikes per sample to achieve the inference.

**Table 7:** Comparison of different models on gas senor dataset.

	**Model**	**T**	**Synapses**	**Accuracy (%)**
Vergara et al. (2013)	SVM	–	–	87.14–96.55
Imam and Cleland (2020)	EPL	16	55.4K	92
**BPSR (this work)**	rMLP	16	**7.7K**	**98.30**

*− The indicator is not applicable*.

## 5. Discussion

SNN promises to realize efficient AI through its brain-inspired mechanism and spike-driven computing architecture. However, the efficiency advantage of the SNN cannot be fully exploited because of the lack of sparsity exploration. This work provides a learning algorithm, namely Backpropagation with Sparsity Regularization (BPSR), to improve efficiency through advanced spiking sparsity and synaptic sparsity. Firstly, a backpropagation algorithm with sparsity regularization is proposed to update parameters and improve sparsity. A heterogeneous LIF neuron dynamics model and a classification loss function with spiking and synaptic regularization are defined. The backpropagation algorithm of the flat and recurrent layer is detailed to calculate the gradient of each parameter. Secondly, the rewiring mechanism based on weight and gradient is proposed to improve synaptic sparsity through pruning and growth. Then, the experimental results show that the proposed BPSR has the advantages of runtime and graphic memory overhead compared with other gradient-based learning algorithms. The improved spiking sparsity can balance the accuracy and FR, and promotes the network performance by simplifying the information representation. Through the BPSR, SNN acquires a structure similar to the nervous system of *C. elegans*, proving its effectiveness. The proposed BPSR reaches the accuracy of 98.33% on the MNIST dataset while achieving 30× inference overhead than other SNN work and 15× energy efficiency compared to BNN after PTQ (with 0.22% accuracy drop). Finally, BPSR is also evaluated on two visual datasets (N-MNIST and CIFAR10) and two sensor datasets (MIT-BIH and gas sensor). The experimental results show comparable or superior accuracy (99.21, 90.74, 98.41, and 98.30%, respectively), with spiking sparsity and synaptic sparsity.

## Data Availability Statement

The original contributions presented in the study are included in the article/supplementary material, further inquiries can be directed to the corresponding authors.

## Author Contributions

YY proposed the idea and did the math and engineering work. YY, HC, and YJ designed the experiments and wrote the first draft of the manuscript. YH, ZZ, and LZ directed the projects and provided overall guidance. ZZ and LZ provided the supervision and project administration. All authors contributed to manuscript revision, read, and approved the submitted version.

## Funding

This work was supported in part by the National Natural Science Foundation of China under Grants 61876039, 62076066, 62004045, and 92164301, Shanghai Municipal Science and Technology Major Projects Nos. 2021SHZDZX0103, 2018SHZDZX01, 17DZ2260900, and NSFC-STINT project No. 62011530132.

## Conflict of Interest

The authors declare that the research was conducted in the absence of any commercial or financial relationships that could be construed as a potential conflict of interest.

## Publisher's Note

All claims expressed in this article are solely those of the authors and do not necessarily represent those of their affiliated organizations, or those of the publisher, the editors and the reviewers. Any product that may be evaluated in this article, or claim that may be made by its manufacturer, is not guaranteed or endorsed by the publisher.
